# Replication-independent change in the frequencies of distinct genome segments of a multipartite virus during its transit within aphid vectors

**DOI:** 10.1128/spectrum.00287-24

**Published:** 2024-03-22

**Authors:** Mathilde Villegas, Michel Yvon, Sophie Le Blaye, Laura Mathieu, Stéphane Blanc, Jean-Louis Zeddam

**Affiliations:** 1 PHIM, IRD, CIRAD, INRAE, Institut Agro, Université de Montpellier, Montpellier, France; Barnard College, Columbia University, New York, New York, USA

**Keywords:** multipartite virus, relative segment frequencies, copy number variation, vector transmission, aphid, plant, nanovirus, virus particle stability

## Abstract

Multipartite viruses exhibit a fragmented genome composed of several nucleic acid segments individually packaged in distinct viral particles. The genome of all species of the genus *Nanovirus* holds eight segments, which accumulate at a very specific and reproducible relative frequency in the host plant tissues. In a given host species, the steady state pattern of the segments’ relative frequencies is designated the genome formula and is thought to have an adaptive function through the modulation of gene expression. Nanoviruses are aphid-transmitted circulative non-propagative viruses, meaning that the virus particles are internalized into the midgut cells, transferred to the hemolymph, and then to the saliva, with no replication during this transit. Unexpectedly, a previous study on the faba bean necrotic stunt virus revealed that the genome formula changes after ingestion by aphids. We investigate here the possible mechanism inducing this change by first comparing the relative segment frequencies in different compartments of the aphid. We show that changes occur both in the midgut lumen and in the secreted saliva but not in the gut, salivary gland, or hemolymph. We further establish that the viral particles differentially resist physicochemical variations, in particular pH, ionic strength, and/or type of salt, depending on the encapsidated segment. We thus propose that the replication-independent genome formula changes within aphids are not adaptive, contrary to changes occurring in plants, and most likely reflect a fortuitous differential degradation of virus particles containing distinct segments when passing into extra-cellular media such as gastric fluid or saliva.

**IMPORTANCE:**

The genome of multipartite viruses is composed of several segments individually packaged into distinct viral particles. Each segment accumulates at a specific frequency that depends on the host plant species and regulates gene expression. Intriguingly, the relative frequencies of the genome segments also change when the octopartite faba bean necrotic stunt virus (FBNSV) is ingested by aphid vectors, despite the present view that this virus travels through the aphid gut and salivary glands without replicating. By monitoring the genomic composition of FBNSV populations during the transit in aphids, we demonstrate here that the changes take place extracellularly in the gut lumen and in the saliva. We further show that physicochemical factors induce differential degradation of viral particles depending on the encapsidated segment. We propose that the replication-independent changes within the insect vector are not adaptive and result from the differential stability of virus particles containing distinct segments according to environmental parameters.

## INTRODUCTION

**Fig 1:**
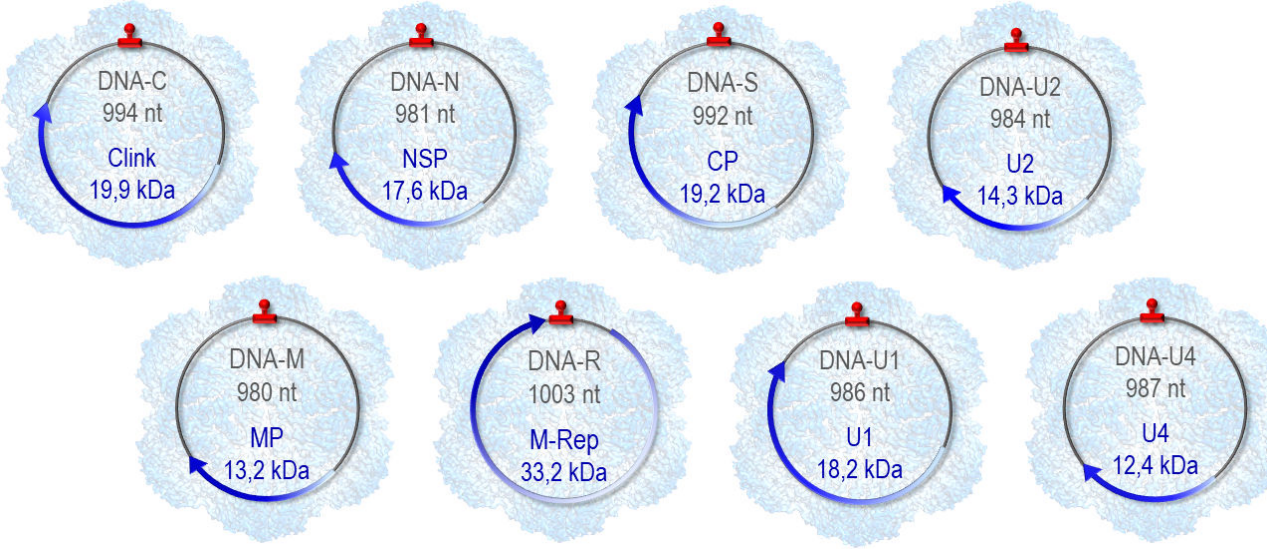
Organization of the FBNSV genome. The eight genome segments, represented by circles, are each individually packaged in a viral particle. The electron density map of the virus particle recently established by Trapani and colleagues (8) is shown in transparent blue in the background. The name of each segment, its size in nucleotides, and the name and size in kDa of its encoded protein are indicated inside the circles. The arrows indicate the position of the open reading frames. A highly conserved region including a stem-loop structure is highlighted in red and corresponds to the origin of replication conserved across all segments.

Viruses can be classified into different categories according to the organization and architecture of their genomic information: monopartite, segmented, and multipartite viruses. Monopartite viruses are composed of a unique nucleic acid molecule protected by a shell, forming the viral particle. Segmented viruses have their genome fragmented into multiple nucleic acid segments, all packaged into a single virus particle. The genome of multipartite viruses is also composed of several nucleic acid segments, but these segments are individually encapsidated in separate viral particles. The nanovirus faba bean necrotic stunt virus (FBNSV; family *Nanoviridae*) is one of the most extreme cases of multiple encapsidation. FBNSV genome is made of eight circular single-stranded DNA segments (Fig. 1), each about 1,000 nucleotides in length and encoding a single protein (1). DNA-C encodes the Clink protein, involved in regulating the host cell cycle; DNA-M encodes the movement protein MP, allowing intra-host movement; DNA-N encodes the protein NSP also named helper component (HC), which does not impact host plant infection but is mandatory for transmission (2–4); DNA-R encodes the protein M-Rep, initiating the replication of all eight viral segments (5); DNA-S encodes CP, the coat protein encapsidating each segment individually; the three U-DNAs (U1, U2, and U4) encode proteins of unknown functions. We have previously established that all eight segments of FBNSV reproducibly accumulate at a specific relative frequency within host plant tissues. This segment-specific frequency pattern varies depending on the host plant species and has been termed “genome formula” (GF) (6). We have also provided evidence for the functional role of inter-host GF modification, enabling the virus to adapt to changing environments by transiently modulating gene expression through gene copy number variation (7).

**Fig 2:**
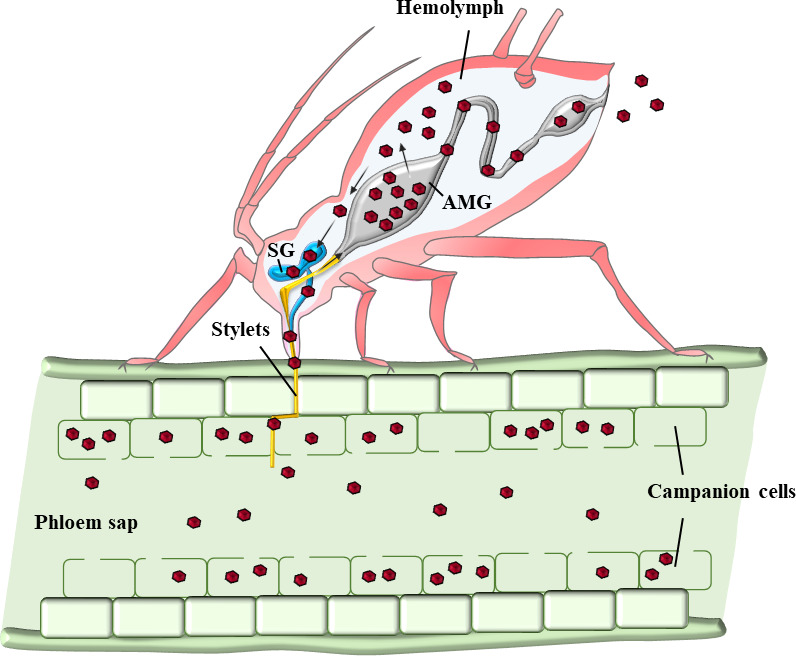
Route of FBNSV particles within the aphid body. Viral particles are ingested by the aphid through the food canal within the stylets bundle and the foregut. Then, they reach the anterior midgut (AMG) and cross the epithelium via a poorly characterized process prior to release into the hemolymph and diffuse to the principal salivary glands (SG). There, they also cross the salivary gland cells and are excreted into the salivary ducts. Finally, particles are inoculated into a new host plant together with the saliva frequently egested during the aphid feeding process. The aphid stylets bundle and foregut are orange, whereas the digestive tract is gray, and the salivary glands are blue. The red hexagons represent the viral particles, and the black arrows indicate their route.

Sicard and colleagues (9) showed that, after virus acquisition, the segment frequencies also changed within aphid vectors. This observation was puzzling because nanoviruses are believed to transit within the aphid body without replicating (10), and so the possible mechanisms inducing a change in the relative frequency of the segments appeared obscure. A similar trend in the changes in the relative segment frequencies was observed in all three aphid vector species tested*, Acyrthosiphon pisum*, *Myzus persicae,* and *Aphis craccivora*, with a notable drop in the segment N frequency and a sharp increase of that of U2. This trend was also similarly observed in the non-vector species *Aphis gossypii* (9). A follow-up study on the route of the virus within the aphid vector (Fig. 2) allowed us to show that (i) all the genomic segments travel together in the individual cells of the vector within numerous cytoplasmic uncharacterized vesicles; (ii) the NSP protein or HC encoded by the N segment is mandatory for the internalization of the virus from the gut lumen into aphid gut cells; and (iii) the viral DNAs perfectly co-localize with the CP and more loosely with the NSP, suggesting that the viral form that transits within the intracellular vesicles of the aphid gut cells is a viral particle-NSP complex (4). Interestingly, a recent structural study indicated that the FBNSV propagates within plant vasculature as virus particles that all have a similar atomic structure, whatever the encapsidated segment (8). This finding indicates that the virus population should be ingested by the aphid together with the plant sap and that any virus particles should be similarly internalized within aphids indifferently of the segment inside.

Here, we further investigate the potential mechanisms that could explain a change in the GF when the virus population is ingested by and internalized within aphids. Sicard et al. (9) initially proposed three possibilities: (i) an unforeseen differential replication of the distinct segments within aphid cells, (ii) a differential internalization of virus particles within gut cells depending on the encapsidated segment, or (iii) a differential degradation of the virus particles containing distinct segment during the viral transfer within the vector. As described above, because viral DNA has only been detected within cytoplasmic vesicles in the aphid gut cells, replication is very unlikely. Indeed, nanoviruses use DNA polymerases from the host plant and replication can only take place in nuclei (11). Likewise, because particles with distinct segments have the same atomic structure, a differential uptake or sorting at gut cell entry appears highly improbable. We thus considered the third possibility, in support of which earlier work on ssDNA bacteriophages reported that particles with the same atomic structure could have distinct properties in solution depending on the encapsidated sequence (12). We first determined where the GF changes actually occur during the route of FBNSV by comparing the relative frequencies of viral segments in different compartments of the vector. The results demonstrate that the only significant changes take place extracellularly, i.e., in the midgut lumen and in the saliva of the aphid. In an attempt to explain the cause(s) of these changes, we carried out experiments that showed that the physicochemical conditions, in particular pH and ionic strength, differentially impact the stability of the viral particles depending on the segments they contain. In itself, this observation can explain the replication-independent relative segment frequencies’ variation within the insect vector, which could thus be a non-adaptive process induced by conditions in the gut lumen and secreted saliva.

## RESULTS

### Change in the relative frequencies of FBNSV segments in the lumen of the anterior midgut of aphids

Previous discoveries have demonstrated a rapid change in the relative frequencies of FBNSV segments when the virus is ingested by aphids (9). For almost a decade, the origin of this phenomenon has been discussed. In the first step, we tried to determine whether these changes occurred within the gut lumen or later, after internalization of the virus within the aphid gut. For this, we compared the relative frequencies of FBNSV segments within the host plants and within aphids fed on these plants, in either the presence or the absence of segment N. It was previously established that when the N segment is omitted, the timing and severity of the symptoms as well as the *in planta* viral load are not affected, while the virus is unable to enter into the vector midgut cells, totally abolishing vector transmission (3, 4).

**Fig 3:**
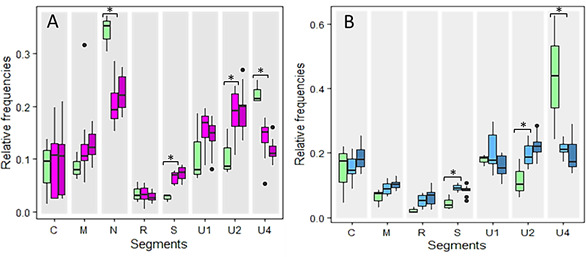
Change in relative frequencies of FBNSV genome segments in aphids. Aphids of the species *Acyrthosiphon pisum* were fed for 3 days on a *Vicia faba* plant infected either with the eight segments of FBNSV (**A**) or solely seven segments with the segment N omitted at agroinoculation (**B**). After this 3-day acquisition period, viruliferous aphids were transferred onto a “healthy” plant. After 24 and 72 h of “purging” on the healthy plant, five pools of five aphids each were collected. The experiment was repeated three times, and the results were pooled to produce the graphs. The relative frequencies of the segments in the source plants were then compared to those within the aphids fed on these plants and purged on healthy plants for 24 and 72 h. Color code in panel A: green for source plants; light purple for aphids at 24 h; and dark purple for aphids at 72 h. Color code in panel B: green for source plants; orange for aphids at 24 h; and red for aphids at 72 h. Standard deviations are represented by circles of the corresponding colors. The asterisks associated with segment names indicate a statistically significant difference between plants and aphids after 24-h purging. The detailed results of the statistical tests are given in the text.

For each of the three experimental repeats, non-viruliferous aphids were deposited on an infected *Vicia faba* plant initially inoculated with all eight segments of FBNSV. Aphid movements were restricted to the apex of the source plants, which corresponds to the area with the highest viral load (6). Following a 3-day acquisition access period, aphids from each individual source plant were transferred to FBNSV-free *V. faba* plants (“healthy plants”) to purge them and eliminate the food bolus. After 24 h of purge, five pools of five aphids were collected from each of these plants for the analysis of the viral content. As reported earlier (9), Fig. 3A shows a change in the relative frequencies of FBNSV segments between source plants and aphids fed on these plants after 24 h of purge (linear model (LM); interaction between organisms and segments, *F* = 3.791, df = 7, and *P* = 9.11e-4). We observed a significant increase in the relative frequencies of segments S and U2 and a decrease for N and U4 (Tukey test; *P* < 0.046).

In order to localize the compartment where these changes occur, we carried out the same experiment using plants infected with an FBNSV missing the N segment, termed hereafter “the N-defective FBNSV” (Fig. 3B). As with plants infected with the full genome, we observed a change in segment frequencies found in aphids after 24 h of purge (LM; interaction between organisms and segments, *F* = 9.819, df = 6, and *P* = 1.18e-8). Remarkably, the segments were affected exactly in the same way as with the full FBNSV genome, with a significant increase of U2 and S and a significant decrease of U4 (Tukey test; *P* < 9.81e-4). Similar changes in the relative segment frequencies were observed whether the virus is internalized in the gut cells (full genome FBNSV) or not (N defective FBNSV), which indicates that the process took place in the gut lumen.

We then wondered if the measured differences in frequencies would increase over time when the virus is not internalized. We collected additional aphids fed on plants infected with the full FBNSV genome or with the N-defective virus after purging for two supplementary days on the healthy plants (72-h purging). We did not detect any difference in the relative frequencies of the segments between 24- and 72-h purging (independent batches), whether the aphids were fed on plants infected with the full genome (LM; interaction between purge time and segments, *F* = 0.664, df = 7, and *P* = 0.702) or with the N-defective ones (LM; *F* = 0.539, df = 6 and *P* = 0.463).

We finally compared the total viral DNA load in aphids after 24 and 72 h of purging, respectively. Surprisingly, the viral load remained stable both when the virus was able to be internalized in the gut cells of the vector (data not shown, LM; effect of purge time on the total DNA load in aphids fed on plants infected with the full set of genome segments, *F* = 0.032, df = 1, and *P* = 0.86) and when it was not able to be internalized (LM; effect of purge time on the total DNA load in aphids fed on plants infected with N-defective FBNSV, *F* = 2.897, df = 1, and *P* = 0.099). We also quantified a similar virus load in aphids fed on infected plants in the presence/absence of segment N, suggesting that the internalized virus particles represented a minute amount of the total virus load within an aphid vector, even after a few days of purging on a healthy plant (LM; effect of the virus internalization in the gut cells on the total DNA load in the aphids, *F* = 0, df = 1, and *P* = 0.997).

Overall, these results demonstrate that the relative frequencies of the FBNSV segments rapidly changed extracellularly, at the entry within the gut lumen, but that such changes were not a continuous process. They occurred during the first 24 h, perhaps immediately, and then the situation appeared “frozen” for the next couple of days, an intriguing observation further discussed later.

We next investigated possible additional modifications in other extracellular compartments, for example, the hemolymph or the saliva of aphids.

### The relative frequencies of FBNSV segments remain stable in aphid hemolymph but change in aphid saliva

**Fig 4:**
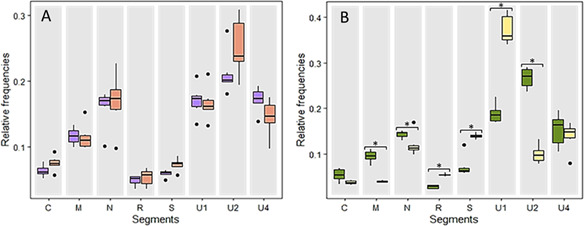
Variation in relative frequencies of FBNSV segments during transit within aphid vectors. (**A**) Aphids of the species *Acyrthosiphum pisum* fed all their life on *Vicia faba* plants infected with the full genome FBNSV were transferred to a “healthy” plant for 24 h. Six pools of 15 anterior midgut (gray) and six pools of 15 hemolymph samples from corresponding aphids (pink) were analyzed and compared. (**B**) Aphids of the species *A. pisum* fed all their life on a *V. faba* plants infected with the full genome FBNSV were allowed to salivate in AP3 nutritive medium through Parafilm membranes for 48 h. Heads (yellow) and saliva (blue) from 20 aphids were collected and pooled. The experiment was repeated six times as indicated in the text, and the results were pooled to produce the graph. Standard deviations are represented by circles of the corresponding colors. Asterisks associated with segment names indicate when the differences in frequencies between the two treatments are statistically significant. The detailed results of the statistical tests are given in the text.

We compared the relative frequencies of the segments between the anterior midgut and the hemolymph from many viruliferous aphids. Adults maintained on FBNSV-infected *V. faba* were transferred to a healthy plant for 24 h and dissected as described in the Materials and Methods section. To ensure that the viral load contained in the hemolymph samples was high enough to be detected by quantitative PCR (qPCR), six pools of 15 aphids were used. Figure 4A shows that there is no significant change in the relative frequencies of the genome segments between the anterior midgut and the hemolymph (LM; interaction between compartments and segments, *F* = 1.671, df = 7, and *P* = 0.128).

**Fig 5:**
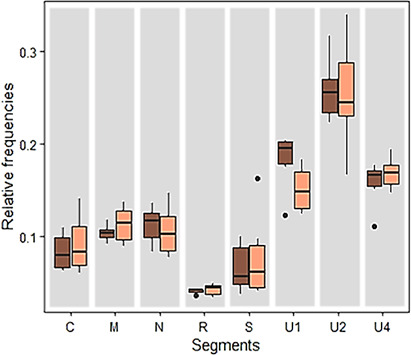
The AP3 medium does not alter the relative frequencies of FBNSV genome segments. A colony of the species *Acyrthosiphum pisum* was maintained on an FBNSV-infected *Vicia faba* plant. The hemolymph of 50 aphids was collected. One part was used pure (purple), and the other part was diluted 20 times in AP3 medium (orange) and then incubated for 48 h. The experiment was repeated six times, and the results were pooled to produce the graphs. Standard deviations are represented by circles of the corresponding colors. No statistical difference appears in the relative frequencies of the segments when comparing viral particles maintained in AP3 medium and those in pure hemolymph (LM; effect of AP3 medium on the relative segment frequencies, *F* = 0.534, df = 7, and *P* = 0.807).

We next compared the relative frequencies of the segments between the salivary glands and the saliva from viruliferous aphids. Aphids fed all their life on *V. faba* plants infected with the full genome FBNSV were transferred to three cylindrical chambers containing AP3 medium (20 aphids per chamber). After 48 h of feeding/salivating, the AP3 from the three chambers were collected and pooled. In parallel, the 60 aphids (20 from each of the three chambers) were dissected to collect the heads containing the salivary glands and pooled. Six repeats of this experiment were performed and used to produce the graphs and statistical analysis (Fig. 4B). The relative frequencies of FBNSV segments significantly changed when the virus was excreted within the saliva (LM; interaction between compartments and segments, *F* = 51.926, df = 7, and *P* < 2e-16). Specifically, the relative frequencies of segments R, S, and U1 were significantly higher in the saliva than in the heads, while M, N, and U2 were significantly lower (Tukey test; *P* < 1.22e-3). We showed that the AP3 medium itself has no effect to confirm that it is the saliva composition that induces the observed change (Fig. 5).

Altogether, our results demonstrate that changes in the relative frequencies of genomic segments take place both in the aphid midgut lumen and in the saliva, two extracellular media where the physicochemical conditions are most likely distinct. Our next effort was then focused on the assessment of putative direct effects of parameters such as pH, ionic strength, or calcium on the FBNSV particles. As mentioned in the Introduction section, these parameters may differentially alter the integrity of virus particles depending on the encapsidated segment and thus differentially promote the degradation of specific segments.

### The stability of FBNSV viral particles containing distinct segments is differentially affected by physico-chemical modifications

**Fig 6:**
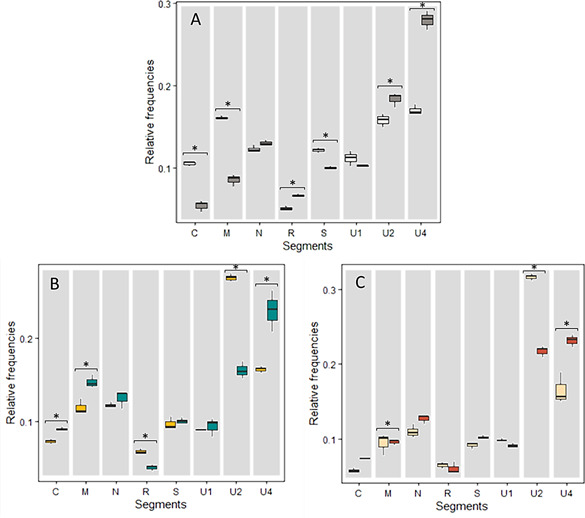
Physicochemical factors differentially impact the stability of FBNSV particles depending on the encapsidated segment. FBNSV viral particles were purified from *Vicia faba* plants and then subjected to different physicochemical conditions. (**A**) The relative frequencies of the segments after treatment at pH = 5.5 and 8.5 are indicated in pink and blue, respectively. (**B**) The relative frequencies of the segments after incubation in 200 mM or 10 mM NaCl are shown in red and yellow, respectively. (**C**) The relative frequencies of the segments before or after incubation with 100 mM CaCl_2_ are shown in green and gray, respectively. All experiments were repeated three times, and the results were pooled to produce the graphs. Standard deviations are represented by circles of the corresponding colors. Asterisks associated with segment names indicate when the differences in frequencies between the two treatments are statistically significant. The detailed results of the statistical tests are given in the text.

We first subjected purified viral particles to different pH based on specific environmental considerations. We tested a basic pH, reflecting conditions of *V. faba* sap (13), and an acidic pH, representing the environment in the gut lumen of *Acyrthosiphon pisum* (14). Subsequently, we applied a DNase treatment to eliminate all non-encapsidated DNA. The DNAse was then inactivated, and the still-protected DNA was extracted and quantified by qPCR. In this experiment, we observed that acidic and basic pH differentially impact the relative frequency of distinct segments (LM; interaction between tested pH and segments, *F* = 82.273, df = 7, and *P* = 2e-16) (Fig. 6A). Specifically, particles containing the segments C, M, and S appeared more stable at the acidic pH, inducing a significant increase in their relative frequency, while it was particles containing R, U2, and U4 that appeared more stable at the basic pH (Tukey test; *P* < 1.5 e-3).

We then similarly tested the effect of ionic strengths and/or distinct salts without *a priori* on the stability of viral particles. First, we examined the effect of a high NaCl concentration (200 mM) on the relative segment frequencies compared to a much lower concentration (10 mM; Fig. 6B). In this case too, we observed a change in frequencies’ distribution (LM; interaction between the two NaCl concentrations and segments, *F* = 34.004, df = 7, and *P* = 1.42e-12). At high ionic strength, particles containing segments C, M, and U4 were more stable, while those containing segments R and U2 proved more labile (Tukey test; *P* < 3.62e-3).

Finally, we subjected the viral particles’ solution to a concentration of 100 mM CaCl_2_ and compared the relative segment frequencies to the initial relative frequencies in the purified viral particles (Fig. 6C). The presence of 100 mM CaCl_2_ affected the relative frequencies of viral segments (LM; interaction between pH tested and segments, *F* = 21.807, df = 7, and *P* = 3.88e-10), with a relative decrease of M and U2 and a relative increase of U4 (Tukey test; *P* < 8.75e-5).

These data prove that physicochemical factors such as those tested here (pH and ionic strength) differentially impact the stability of FBNSV viral particles according to the encapsidated genome segment and that this phenomenon can induce an apparent change in the FBNSV GF.

## DISCUSSION

The term GF was initially coined for FBNSV to describe the fact that the relative frequencies of viral segments rapidly reach an equilibrium during infection (6). The FBNSV GF is highly reproducible in a given plant species but immediately and reversibly changes when the virus switches hosts. Additional investigations conducted on other multipartite viruses have shown that their genomic segments also accumulate at different frequencies in the host, yielding a genome formula that is also host dependent (15–18). Although this phenomenon seems to be widespread among multipartite viruses (19), the mechanisms underlying the establishment of the genomic formula remain an enigma. Both theoretical and empirical attempts at unraveling such mechanisms suggest that they are likely related to within-plant differential replication of individual segments or of groups of segments in order to adapt to distinct environments (7, 20). As indicated in the Introduction, a similar mechanism could hardly explain the change in frequencies of the FBNSV segments within the aphid vectors, where FBNSV does not replicate.

Consistently, using a helper (N)-defective FBNSV that particles are not able to internalize within the vector gut cells (4), we here show that the first change in the relative segment frequencies occurs in the gut lumen, i.e., when the virus is still extracellular. This represents a substantial advancement over Sicard et al. (9), who solely examined aphid whole bodies. We also show that most of the virus present within the aphid is durably located in the midgut lumen (which means that the measured segment frequencies are defined by the non-internalized virus) as the total DNA loads are similar in aphids fed on plants infected with all eight segments and those fed on the N-defective ones after a purge period of 24 h. Because FBNSV particles generated upon infection by the N-defective FBNSV are not internalized in aphid gut cells (4), they cannot be transmitted, and they are not possibly inoculated to the healthy plant during the purging period. Our data thus established that both the relative segment frequencies and the total DNA amount are similar in aphids sampled after two purging periods (24 and 72 h, respectively) using either the wild-type virus or the N-defective one. Because viruses transmitted by hemipteran vectors in a circulative non-propagative manner (just as is FBNSV) are known to cross the gut barrier within a few hours (21–23), it was a surprise to find that the virus can persist for at least 72 h in the gut lumen. We here hypothesize that FBNSV particles are internalized within gut cells at a slow rate and that most of them remain trapped in the mucus secretions abundantly produced in the lumen (24), where they can be protected and stored for several days. This may explain why the early changes in the FBNSV segment frequency visible in aphids after 24 h purging do not further progress in aphids collected 2 days later. This hypothesis is also consistent with the earlier demonstration that FBNSV virus particles can “wait” for the helper component NSP for at least 48 h before being internalized and transmitted (25). However, the specific details of these mechanisms and the interactions between viral and aphid components both in the lumen and upon internalization have yet to be elucidated.

When comparing the relative segment frequencies between the midgut and the hemolymph, no noticeable change was identified. These results are in agreement with a previous work, which demonstrated that the frequency distributions of the segments are similar between the midgut and the head of aphids (9). In contrast, when comparing the relative segment frequencies between aphid heads and secreted saliva, we identified a significant change for six of the eight genomic segments. Here too, a differential replication of FBNSV segments specifically within the salivary glands could hardly explain the observed changes since no difference could be detected between the midguts and the heads of aphids (9). Nevertheless, we cannot totally exclude that a small proportion of the viral population could replicate, as it has been demonstrated for the begomovirus tomato yellow leaf curl virus that an elusive transient replication phase occurred in the salivary glands of its whitefly vector, shortly after acquisition (26, 27). Another hypothesis could be that segments would be differentially released into the salivary ducts. This is not consistent with the observation that no change occurs in the relative frequencies of the segments when internalized in and released from the midgut, nor when they are internalized in the salivary gland cells. The fact that particles containing distinct segments most probably have the same capacity to enter and exit aphid cells is supported by two arguments: (i) the internalization of particles is not direct but is mediated by the helper factor NSP, which is likely the viral factor interacting with aphid putative receptors (4); (ii) we recently solved the atomic structure of the FBNSV particles and could not detect any structural differences between particles containing distinct segments (8). Thus, there is no structural ground that could allow particle sorting depending on the segment inside, and so we believe that differential internalization or release from gut and salivary gland cells is highly unlikely. Our preferred alternative explanation is that the physicochemical conditions differentially impact the stability of the viral particles depending on the encapsidated segment, as this could similarly explain the segments’ frequency changes in the gut lumen and in the saliva.

We thus assessed this hypothesis and demonstrated that the pH, the ionic strength, and the ions present in the solution induce modifications in the relative frequencies of the different segments of populations of purified viral particles. The cryo-electron microscopy reconstruction of the 3D structure of the FBNSV virus particles at 3.2-Å resolution did not identify major structural differences depending on the packaged segment (8). Yet, our results clearly demonstrate that the properties in solution of particles containing different FBNSV segments are not strictly identical. The packaging uses ssDNA segments as a scaffold enabling CP conformational changes, particularly in the N-terminal part that interacts with the DNA within virions. Our recent structural analysis of FBNSV particle (8) revealed 60 putative inner contact points, one per CP subunit, between the DNA and the coat protein. Being single stranded, segments may adopt variable secondary structures, with variable proportions of stems and loops, depending on their sequence and on local physicochemical conditions. Distinct secondary structures may allow to attach variable proportions of these 60 putative contact points through negative charges, and thus some segments might consolidate the viral particle more efficiently. Although this specific aspect has never been investigated, it likely affects particle stability (12). Unfortunately, ssDNA secondary structure predictions cannot account for what happens when the distinct DNAs interact with the inner contact points inside particles, which may vary with distinct pH, ionic strength, and different divalent cation contents. It is thus impossible to predict the number of contact points that will effectively be connected by a given segment within a viral particle in a specific environment, precluding any prediction of particle stability and genome formula variations within aphids.

Beyond pH and ionic strength, it would have been interesting to verify whether other physical (e.g., temperature) or biochemical (e.g., proteases) factors could also induce differential degradation of FBNSV particles, as earlier evidenced for a tripartite virus (28, 29). Unfortunately, given the difficulties encountered in purifying FBNSV and the limited quantities obtained, we were unable to carry out additional tests.

Previous work has demonstrated that the relative frequencies of the genome segments of faba bean necrotic yellow virus (FBNYV), a nanovirus closely related to FBNSV (30), also vary when ingested by *A. pisum* from infected *V. faba* host plants (18). Although there is a high level of homology between FBNSV and FBNYV genomes, some segments do not behave the same way in the aphid vectors; for example, particles containing the U2 segment of FBNSV increase in frequency within insects, while those of FBNYV remain constant. In contrast, particles containing the N segment of FBNSV are labile, while those of FBNYV are more stable in aphids. All other factors being equal to those of our experiments with FBNSV, these results point out to the variation in the genome sequence, and its derived products, i.e., capsid, being at the origin of the differences in the stability of the viral particles.

In conclusion, our results show that a change in the relative frequency of the distinct segments of FBNSV quickly takes place when the viral particles encounter environments with drastically different physicochemical conditions. This is particularly the case in the gut lumen and in the saliva of aphids, where both pH and ion content (and also redox state and enzymes) are very different from the intracellular context (14, 31). We hardly see how such a phenomenon could represent a functional process as is the case in the host plant (7), and we thus predict that changes in the nanoviruses’ relative segment frequencies within their aphid vectors are a fortuitous non-adaptive effect. This phenomenon could also be present in vectored multipartite viruses belonging to other families.

## MATERIALS AND METHODS

### FBNSV isolate, host plants, and inoculation

FBNSV was first isolated in Ethiopia from faba bean plants (32) and maintained in the lab by successive passages through aphid transmission. A few years later, an infectious and aphid-transmissible clone was constructed and characterized (33). Each of the eight genomic DNAs was cloned independently, as a tandem repeat, into plasmid pBin19 and transferred into *Agrobacterium tumefaciens* strain COR308. Plasmids were inoculated into plants as a mixture of bacteria cultures as previously described (6). In laboratory conditions, some segments are dispensable and can be omitted at inoculation without compromising the plant’s systemic infection. In particular, when the N segment is omitted, the timing and severity of the symptoms as well as the *in planta* viral load are not affected, while the virus is unable to enter into the vector midgut cells, totally abolishing vector transmission (3, 4).

Faba bean plants (*Vicia faba*; var. “Sevilla,” Vilmorin) were sown with one seed per pot. The plants were maintained in a growth chamber with 70% hygrometry, a photoperiod of 13 h day time at 26°C and 11 h night time at 20°C. Ten-day-old *V. faba* seedlings were agro-inoculated with the infectious clone of FBNSV. Twenty-one days post-inoculation, symptomatic plants were analyzed by quantitative PCR to determine the presence/absence and quantify each inoculated segment.

### Aphid rearing and dissection

Some colonies of the aphid clone LL01 of the species *Acyrthosiphum pisum* were maintained on healthy plants and others on FBNSV-infected plants. Aphid colonies were reared under controlled conditions, with a photoperiod of 13 h day time at 25°C and 11 h night time at 19°C. These conditions ensure reproduction by parthenogenesis. The hemolymph of aphids was collected by removing aphid antennae and cornicles and pipetting the exuding droplets with a 10 µL micropipette tip. The anterior midguts and heads were dissected in 1× phosphate-buffered saline (pH 7.4). Aphid saliva was sampled by transferring aphids in cylindrical chambers topped with artificial medium “AP3” (34) maintained between two Parafilm membranes. After 48 h of feeding, the medium containing the secreted saliva was collected (35). All samples were stored at −20°C until processed.

### Purification of virus particles and stability test

FBNSV particles were purified from 100 grams of virus-infected *V. faba* apices as previously described (8) and stored at 4°C. Next, the virus particles were transferred to different buffers varying in pH and ionic strength, using two distinct types of salts. The composition of the distinct buffers is indicated in Table 1. Purified virus particles were diluted 20-fold in each buffer and incubated for 2 h at 20°C. To eliminate free DNA released from the disassembled particles, we used the RQ1 RNase-Free DNase (Promega) according to the manufacturer’s instructions. This DNase was then heat inactivated using the provided stop solution. The relative frequency of particles containing distinct segments that resisted the treatment, thus protected from the DNase treatment, was finally estimated by qPCR.

**TABLE 1:** Composition and pH of the different buffers used to test the differential stability of FBNSV particles

Buffer	Added salt	pH
20 mM Tris-HCl	–	5.5
20 mM Tris-HCl	–	8.5
20 mM Tris-HCl	NaCl 10 mM	7
20 mM Tris-HCl	NaCl 200 mM	7
20 mM Tris-HCl	CaCl_2_ 100 mM	7

### Viral DNA extraction

For all plants used, total DNA extraction was performed as previously described (4, 25). Briefly, three different leaves located immediately below the apex of infected plants were squashed onto a Whatman paper disk of 0.6 cm diameter. Each disk was individually deposited onto the filter of a 200 µL micropipette tip. Then, 100 µL of modified Edwards buffer [200 mM Tris-HCl pH 7.5, 25 mM EDTA, 250 mM NaCl, 0.5% SDS, 1% polyvinylpyrrolidone 40 (PVP40), and 0.2% ascorbic acid] was added and centrifugation at 5,000 *g* for 15 s was performed directly into a PCR plate placed underneath. Finally, DNA was precipitated with 50% isopropanol (final concentration), rinsed with 70% EtOH, and resuspended in 30 µL nuclease-free H_2_O. Pestle-crushed whole aphids and dissected heads or guts, hemolymph, AP3 medium, and purified virus samples were extracted using the Purelink Plant Total DNA Purification Kit (Invitrogen) according to the manufacturer’s instructions. DNA was resuspended in 70 µL nuclease-free H_2_O.

### Quantitative real-time PCR detection

Total DNA extracts from plants were diluted 10-fold, while purified virus and aphid extracts (anterior midguts, heads, hemolymph, saliva, and AP3 diet) were used undiluted. qPCR quantification was performed using the LightCycler FastStart DNA Master Plus SYBR Green I kit (Roche) following the manufacturer’s instructions. The total reaction volume per well was 10 µL, consisting of 5 µL of 2× qPCR Mastermix, 0.3 or 0.5 µM primers [0.3 µM final for C, M, and S segments and 0.5 µM for other segments; FBNSV segment-specific primers have been described elsewhere (6)] supplemented to 8 µL with H_2_O, and 2 µL of DNA extract. Forty qPCR cycles of 95°C for 10 s, 60°C for 10 s, and 72°C for 10 s were applied using the LightCycler 480 thermal cycler (Roche, Indianapolis, IN, USA). Each sample was run in duplicate. Post-qPCR data were analyzed and then converted to nanograms of DNA using standard curves, as previously described (36). The relative frequency of each genome segment was obtained by dividing the estimated quantity of one given segment by the sum of those of all eight, as explained in Sicard et al. (6).

### Statistical analysis

All statistical analyses were performed using R software, version 4.3.1 (2023; R Development Core Team). To assess whether there was a change in the relative segment frequencies between FBNSV-infected plants and aphids fed on these plants and purged for 24 h on a healthy plant, a linear model was applied with the *lm* function from the stats package taking into account the effect of experimental repetitions. Then, an ANOVA was done to test the interaction of plant or aphid compartment and segments’ effects (Fig. 3A and B). A similar approach was run to test (i) the effect of purge time on the DNA amount detected in aphid anterior midguts (data not shown), (ii) the effect of purge time on the relative frequencies of FBNSV segments (Fig. 3A and B), (iii) the effect of the AP3 medium on the relative segment frequencies (Fig. S1), (iv) whether there was a change in relative segment frequencies between aphid anterior midguts and hemolymph samples (Fig. 4A), (v) whether there was a change in relative segment frequencies between aphid heads and saliva (Fig. 4B), and (vi) whether there was a change in relative segment frequencies under the effect of different pH and ionic strengths/salts added in solution (Fig. 6A through C). A *P-*value of <0.05 was considered statistically significant. In this case, two by two comparisons were carried out with a Tukey test with *ghlt* function and the multcomp package. The statistical tests used and the associated results are mentioned throughout the text.
